# Emerging paradigms in microwave imaging technology for biomedical applications: unleashing the power of artificial intelligence

**DOI:** 10.1038/s44303-024-00012-8

**Published:** 2024-06-03

**Authors:** Nazish Khalid, Muhammad Zubair, Muhammad Qasim Mehmood, Yehia Massoud

**Affiliations:** 1 Department of Electrical Engineering, Information Technology University of the Punjab, Lahore, Pakistan; 2 Innovative Technologies Laboratories (ITL), King Abdullah University of Science and Technology (KAUST), Thuwal, Saudi Arabia

**Keywords:** Medical imaging, Biomedical engineering

## Abstract

In recent years, microwave imaging (MWI) has emerged as a non-ionizing and cost-effective modality in healthcare, specifically within medical imaging. Concurrently, advances in artificial intelligence (AI) have significantly augmented the capabilities of medical imaging tools. This paper explores the intersection of these two domains, focusing on the integration of AI algorithms into MWI techniques to elevate accuracy and overall performance. Within the scope of existing literature, representative prior works are compared concerning the application of AI in both the “MWI for Healthcare Applications" and “Artificial Intelligence Assistance In MWI" sections. This comparative analysis sheds light on the diverse approaches employed to enhance the synergy between AI and MWI. While highlighting the state-of-the-art technology in MWI and its historical context, this paper delves into the historical taxonomy of AI-assisted MWI, elucidating the evolution of intelligent systems within this domain. Moreover, it critically examines prominent works, providing a nuanced understanding of the advancements and challenges encountered. Addressing the limitations and challenges inherent in developing AI-assisted MWI systems like Generalization to different conditions, Generalization to different conditions, etc the paper offers a brief synopsis of these obstacles, emphasizing the importance of overcoming them for robust and reliable results in actual clinical environments. Finally, the paper not only underscores the current advancements but also anticipates future innovations and developments in utilizing AI for MWI applications in healthcare.

## Introduction

We currently find ourselves in the era of algorithms, where Machine Learning (ML)/Deep Learning (DL) systems have significantly transformed numerous industries, including development, transportation, and regulation, as well as healthcare, making them an integral part of our everyday existence^1^. The convergence between AI and healthcare has paved the way for significant medical diagnostics and treatment progress^2^. The availability of large datasets and advancements in computational techniques have empowered AI to augment accuracy and efficiency across diverse medical imaging modalities^3^. Within this context, MWI is a cost-effective technology has captured considerable attention for its prospective applications within biomedical imaging^4,5^.

MWI has emerged as a highly promising modality for medical imaging, attracting considerable focus in recent years due to the development of imaging algorithms and data-collection hardware^5^. The implementation of MWI medical devices has increased researchers’ devotion and they started significant efforts to investigate this topic extensively. worldwide in the last two decades^6^, especially for medical applications^7^. The MWI methodology involves solving an electromagnetic inverse scattering problem to reconstruct the distribution of tissues that possess different electrical properties^8^. Medical imaging using MWI has primarily been utilized for breast cancer screening, diagnosing cerebrovascular diseases, treatment, monitoring, and the progression of disease i.e. Alzheimer’s disease^5^. It also aids in other healthcare applications, including nondestructive testing such as Nondestructive Evaluation for inspection of biological bodies^9^, Non-invasive analysis, and detailed imaging of biomaterials and biological tissues^10^, and thermal therapy systems^11^.

Due to its unique advantages, MWI differs from traditional imaging methods such as X-ray, ultrasound, or magnetic resonance imaging (MRI). It is exemplary because it can effectively penetrate biological tissues, is highly sensitive to tissue dielectric properties, provides high contrast resolution^12^, offers a noninvasive approach to examining functional and pathological characteristics within soft tissues^13^, and avoids exposing patients to ionizing radiation, which can be detrimental to health^14^. Additionally, the affordability and small size of MWI devices contribute to enhanced accessibility for various medical facilities^12^. These inherent qualities make MWI particularly suitable for different applications such as early-stage cancer detection, monitoring therapy response, and assessing tissue composition^13^.

Although MWI techniques have achieved remarkable outcomes, recent research in this domain has identified several challenges and limitations, such as the limited spatial resolution compared to other modalities, which can make it challenging to accurately discern intricate details about diminutive objects or structures^15^, diffraction limits imposed on microwaves, which make it difficult to identify fine details about small objects or structures precisely in far-field^15^, real-time imaging capabilities remain elusive and need data interpretation as ultimately generates substantial data requiring accurate analysis and interpretation^16^.

AI has reshaped the healthcare domain, presenting itself as an influential force that allows for a thorough examination of complex medical data quickly and precisely^17^. Unprecedented benefits have been observed when MWI is paired with AI techniques, showing immense potential for the future. With the intelligent application of AI algorithms, MWI can effectively tackle common obstacles such as noise interference, scattering complications, and restricted spatial resolution, ultimately improving the overall quality of reconstructed images^5^. Furthermore, AI technology has emerged as an invaluable aid in facilitating timely decision-making processes, automating image interpretation tasks, and delivering essential insights to clinicians and radiologists. Researchers are actively exploring various AI techniques encompassing DL^18–20^, ML^21^, to bolster the performance of MWI. The successful application of these techniques thus far is evident through their positive impact on important tasks such as image reconstruction, feature extraction, and classification. These improvements positively influence diagnostic accuracy and ultimately lead to better patient outcomes.

The convergence of two cutting-edge technologies, MWI and AI, has given birth to an unprecedented transformation in biomedical imaging research^22,23^. This amalgamation is paving the way for a remarkable renaissance in healthcare diagnostics by offering unparalleled prospects for accurately evaluating different medical conditions through non-invasive means in real-time. Through this paper, we aim to explore the fascinating fusion of MWI and AI while illuminating their immense potential to reshape the field of medical imaging and propel us toward a groundbreaking era in healthcare innovation. This study provides a complete literature review of AI-assisted MWI for healthcare applications, focusing on the most recent MWI and AI methodologies and the areas of overlap between these two fields. The fundamental goal of this work is to provide readers with a complete grasp of current state-of-the-art MWI methodologies and the usage of AI in this sector, along with possible solutions to the difficulties encountered during this process in the form of implementation. The lack of research in this area is also discussed. The salient contributions that this work makes are summarized here.

1. We formulate an overview of diverse literature on the state-of-the-art MWI techniques in the healthcare domain by categorizing each technique and its application in healthcare, i.e., passive, active, and hybrid MWI techniques.
2. We provide a comprehensive overview of the currently available AI-assisted MWI methodologies, expanding on each method while keeping the healthcare industry as our primary research focus. The taxonomy of AI-assisted MWI is broken down in great detail here.
3. Finally, we identify various unresolved research topics that call for additional inquiry and challenges. Provides in detail limitations and future directions.

In comparison to related MWI studies, our paper stands out for its comprehensive exploration of AI-assisted MWI in healthcare. While existing reviews touch on specific applications such as lung tumor detection^24^, breast cancer^25^, and brain stroke^26^, our paper uniquely focuses on healthcare as a whole. We extensively discuss various MWI techniques, incorporating both ML and DL algorithms. Unlike some reviews that only partially address AI integration, our paper consistently emphasizes the application of AI, offering a detailed survey of techniques, challenges, and future directions. Notably, our work excels in presenting a holistic view of MWI in medicine, encompassing improved imaging and diverse medical applications. This breadth of coverage positions our paper as a comprehensive guide, providing valuable insights for researchers, practitioners, and stakeholders interested in the intersection of AI and MWI in healthcare.

## Search approach

A comprehensive search was conducted across multiple databases, including Google Scholar, PubMed, and IEEE Xplore, with no restrictions on language or time. The focus of the literature search for detection, and prediction techniques was on the application of microwave imaging in healthcare and novel methods incorporating machine learning models. The search criteria utilized the keywords: “(Neural networks OR deep learning OR artificial intelligence OR machine learning OR clinical diagnosis OR prediction) AND tumor detection and treatment." Regarding the utilization of microwave imaging, the literature search emphasized diagnostic applications. The search criteria involved the keywords: “(tumor detection, stroke detection OR microwave imaging OR dielectric properties of tissues OR tissue classification OR microwave ablation OR microwave radiometry OR AI-assisted diagnostics) AND microwave imaging in healthcare." A thorough research, barring any time or language limit was conducted, on Google Scholar, PubMed, and IEEE Xplore. The focal point of this scavenge was to identify the application of Microwave Imaging in healthcare and novel models that incorporate Machine Learning Algorithms to improve detection and prediction. The following keywords were used in the search criteria: “(Neural networks OR deep learning OR artificial intelligence OR machine learning OR AI assisted Microwave Imaging OR clinical diagnosis OR prediction) AND tumor detection and treatment." In the context of microwave imaging applications, the literature search targeted diagnostic applications. The keywords used in the search were: “(tumor detection, stroke detection OR microwave imaging OR dielectric properties of tissues OR tissue classification OR microwave ablation OR microwave radiometry OR AI-assisted diagnostics) AND microwave imaging in healthcare."

## MWI for healthcare applications

### MWI techniques

**Fig. 1:**
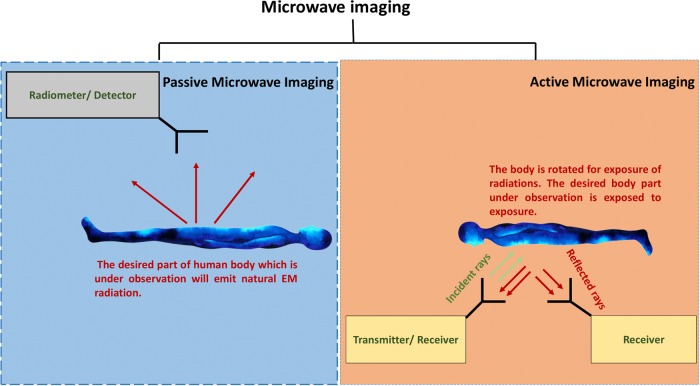
Working principle of MWI techniques.

This section provides a comprehensive overview of the essential techniques used in MWI for biomedical applications. MWI encompasses three primary methods, Passive, Active, and Hybrid, each described in detail below. Figure 1 shows the working principle of MWI techniques^27^.

#### Passive MWI

Passive microwave radiometry (MWR) is a technique that measures natural emissions between 1 to 10 GHz. Cells, proteins, organs, and even the whole body are all appropriate targets for this method. What’s particularly interesting is that biochemical and biophysical processes determine the strength of the intrinsic emission. In contrast to infrared thermography (IRT), which can only detect emissions several microns deep (skin temperature), MWR can detect thermal abnormalities as shallow as several centimeters measure internal/deep temperature. MWR is a low-cost method that has no negative effects on human health. Ionizing radiation or any other form of radiation and fluorescent or radioactive tagging is neither required nor necessary. MWR can be useful in preclinical and clinical research and the early stages of the drug discovery process^28^.

#### Active MWI

Active MWI techniques involve inundating the area to be inspected with electromagnetic signals operating at microwave frequencies, sensing and collecting the energy reflected or scattered by the part, and then using this data to form images. Tomography and radar-based imaging are the two main types of active MWI.

#### MWI tomography

Microwave tomography (MWT) provides two-dimensional slices or images of an object of interest (OI) using its dielectric properties by measuring the changes in the electromagnetic field. In most MWT techniques, antennas surround the OI in an imaging chamber. Filling the imaging chamber with a substance that connects the majority of the microwave electromagnetic energy to the affected region increases the performance of the system. While testing, every antenna will emit clockwise electromagnetic signals of one or more frequencies. Due to dielectric property differences between the OI and the matching medium and inside the OI, additional non-transmitting antennas measure electromagnetic fields. The data is processed into specialized algorithms, which provide 2D visualizations of the dielectric characteristics^29^.

#### Radar-based MWI (RBMWI)

The radar-based MWI uses the reflected signals collected due to the sudden variations in the electrical properties of the OI material to construct the image. The MWT, on the other hand, quantifies signals resulting from the overall OI material change^30^. There are currently five categories that can describe developed radar-based MWI systems^31^, namely: Confocal MWI (CMI)^32^, Multi-static adaptive (MSA) system^33–35^, Tissue-sensing adaptive radar^36,37^, MWI via space-time (MIST) beamforming^38^, and Holographic MWI (HMI)^39^.

#### Hybrid MWI

The goal of the design process for hybrid techniques is to combine the benefits of multiple MWI approaches into a single solution. Fundamentally, it’s based on combining two different methods to achieve better outcomes. Some famous hybrid techniques are explained herein.

### History of MWI in healthcare contribution in clinical techniques

The contribution of E. Larsen and J.H. Jacobi in the 80’s can be seen as the catalyst for a significant research and development endeavor focused on near-field MWI techniques related to Industrial, Scientific, and Medical applications. The initial work of this domain is the construction of remarkable images of isolated perfused dog kidneys introducing a fresh perspective on microwave imagery, which had previously been primarily directed toward far-field radar target imaging^40^. In the 1980s, Rosen discusses 6 Giga Hertz (GHz); microwave thermography used to detect breast cancer. An evaluation of the antenna power pattern was done, and it showed that almost all the energy received at 6GHz originates within the 3 mm skin layer overlying the breast. Second, that resolution is likely to be limited more by examination time than by the intrinsic power pattern of the antenna^41^. In the 1980s, the MWI applications in healthcare increased like diagnostic^42^, and medical applications^43^. In the 1990s, MWI has undergone significant advancement. Embracing both microwave tomography and radar-based imaging. Moreover, extensive research and clinical trials have been conducted to enhance the precision and dependability of microwave breast imaging.

Computer-aided detection (CAD) systems have also been introduced to facilitate interpreting MWI results. The noticeable work in that decade includes tissue blood content changes^44^, tissue assessment^45^, new approaches for breast cancer detection^46–48^, ex vivo breast cancer experiments with MWI^49^, MWI camera for image reconstruction for biomedical applications^50^, microwave tomographic scanner^51^. In the 2000s, breast cancer detection, ongoing progress, and MWI technology enhancement were observed. Numerous clinical studies and trials have been conducted to evaluate the efficacy and safety of microwave breast imaging thoroughly. Furthermore, researchers are exploring the potential applications of MWI for other important areas, as seen in the 1990s. In 2000 for the first time, an active clinical MWI-based prototype was designed to report that a clinical prototype of an MWI system had been developed. Using a 16-element transceiving monopole antenna array of 300–1000 MHz this system illuminates the breast^52^.

In this score years, much work has been done for MWI applications, such as breast cancer detection. New and better approaches are proposed, like MWI for breast cancer detection and 3-dimensional tumor localization^53^, MWI for breast cancer detection via space-time^54^, 3-dimensional MWI breast cancer screening^55^, Active 3-dimensional MWI prototype and experiment setup^56^. Not only breast cancer but researchers also move to other organ imaging like the brain for stroke detection and heart imaging. Semenov et al.^57^ investigate whether alterations in dielectric properties could indicate physiological changes in the myocardium of canines resulting from a reduction in coronary blood flow, infarction, and ischemia. Semenov et al.^58^ conducted a study on the dielectric properties of canine myocardium during acute ischemia and hypoxia.

The feasibility of microwave tomography for detecting myocardial infarction is being evaluated by examining the variations in dielectric properties between normal and infarcted tissues. Images were obtained from excised canine hearts that had experienced myocardial infarction for two weeks^59^. Also, other applications, like conducting a research study in developing microwave tomography for functional cardiac imaging^60^, three-dimensional MWI for biomedical applications^61^, microwave tomography for bone imaging^62^, 3-dimensional Bone imaging^63,64^, neoadjuvant chemotherapy monitoring^65^, Differential MWI for brain stroke^66^, lung detection using frequency MWI^67^, lungs’ detection using MWI^68^, and human Forearms: Pilot Study and Image Enhancement using MWI^69^. Many improved MWI-based biomedical equipment has been designed to advance the medical care system in the last five years. Islam et al.^70^ developed a novel, efficient, and affordable MWI system for breast imaging. To enhance the performance of this system, the author has employed an iterative enhancing technique. Furthermore, the author has designed a compact side-slotted tapered slot antenna specifically for MWI. SAFE (Scan And Find Early) is an MWI device designed specifically to screen and detect breast cancer at its early stages. By utilizing completely safe electromagnetic waves, SAFE offers valuable preliminary diagnostic information. Eliminating the need for X-rays^71^. Moloney et al.^72^ proposed the first-in-human clinical investigation of the Wavelia System^72^.

**Fig. 2:**
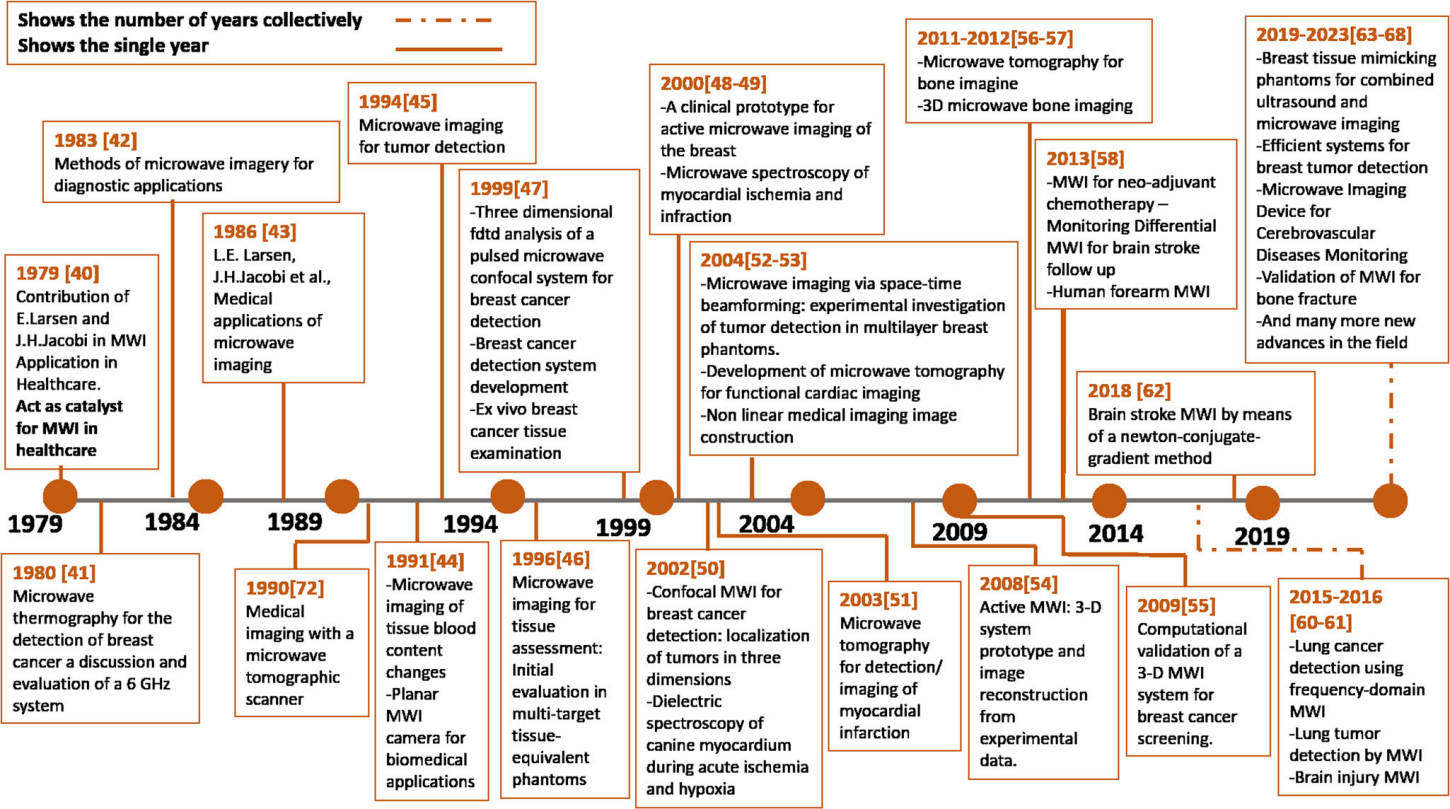
Taxonomy of Evolution of MWI techniques in Biomedical Domain. Legends : 1979^40^, 1980^41^, 1983^42^, 1986^43^, 1991^44^, 1994^47^, 1996^45^, 1999^48^, 2000^52^-^57^, 2002^58^, 2003^59^, 2004^60,61^, 2008^56^, 2009^55^, 2011^62^, 2012^63^, 2013^65,69^, 2015^67^, 2016^68^, 2018^64^, 2019–2023^70–76^.

The use of MWI to detect fractures in superficial bones such as the tibia using a straightforward and practical setup is worth considering due to its feasibility^73^. The validation of an experimental MWI system for real-time monitoring of brain stroke in the post-acute stage is necessary^74^. The main objective is to develop, implement, and verify a comprehensive device for detecting and monitoring cerebrovascular diseases through microwave technology. The created system is a compact and simple prototype with a wearable antenna array of twenty-two elements. This array enables continuous real-time monitoring of the progression of a brain stroke^75^. Santos et al.^76^ offer a systematic assessment of the efficacy of an air-operated MWI system in detecting thin fractures in superficial bones such as the tibia. Moreover, it introduces a novel-conveniently sized setup featuring a single Vivaldi antenna that conducts a semi-cylindrical scan of the limb. Ongoing research efforts by scientists involve investigating advanced imaging algorithms and refining hardware designs for improved accuracy, resolution, and clinical applicability in MWI. Furthermore, there is a focus on integrating this technology with other modalities used for medical imaging purposes. The successful integration of MWI into routine medical practice would vastly enhance personalized patient care through its potential benefits. Figure 2 shows the taxonomy of the prominent history of the MWI technique’s contribution to the healthcare domain.

### State of Art MWI techniques for disease detection

#### Bones

Alkhodari et al.^77^ proposed human bone density monitoring using MWT. Using the reconstruction approach, the human bone representations of different thicknesses were developed. Based on the results of this research, MWT shows its excellent potential as a bone imaging modality because it employs non-ionizing radiations to produce high-quality images. Laskar et al.^78^ study’s overarching goal was to determine not joint temperatures recorded using the quick and simple MWR technique may indicate inflammation without obvious clinical symptoms. In evaluating individuals with rheumatoid arthritis and spondyloarthritis, the author expected that MWR might prove useful. Subclinical and overt inflammation is reflected in elevated MWR-derived joint temperature, suggesting its potential use as a biomarker in arthritis. Goryanin et al.^28^ explain in detail the biomedical applications of MWR.

#### Skin

Owda et al.^79^ presented using millimeter-wave (MMW) passive radiometry as a non-contact sensor that could detect skin diseases and disorders without requiring invasive procedures. The author used 90 GHz radiometry to measure the reflectance of two groups of people: one group of 60 people with healthy skin, with an equal ratio of males and females, and another group of 60 people with skin ailments and diseases such as basal cell carcinoma (BCC), squamous cell carcinoma (SCC), burn wounds, and eczema. Radiometry studies demonstrate a considerable difference in reflectance comparing fine and afflicted skin, ranging from 0.02 to 0.27. Based on these results, non-contact sensing techniques like radiometry may detect and differentiate between healthy and diseased skin. MWR could be utilized to detect skin diseases and anomalies without causing any harm to the patient.

#### Brain

Amin et al.^80^ proposed experimental phantoms of healthy and diseased human calcaneus bones that were used to collect data. The research focuses on the distorted born iterative method (DBIM) algorithm and its application in monitoring skeletal health. This research is the first technique to employ MWI for imaging bone health. Henriksson et al.^81^ proposed a portable electromagnetic tomography brain scanner prototype for human clinical studies. The scanner was reliable and safe enough for use in a clinical investigation. The preliminary study involving healthy participants and stroke patients who underwent testing with a mobile electromagnetic scanner was successful. The study aimed to demonstrate the technology’s clinical safety and effectiveness in identifying ischemic from hemorrhagic strokes. The scanner’s efficacy and safety were confirmed in non-healthy and healthy patients, with preliminary research suggesting it can spot brain damage in stroke victims. Goryanin et al.^82^ proposed preliminary egg white microwave emission investigations during ethanol-induced denaturation. The author discovered that microwave emissions changed without changing water thermodynamic temperature. Thermodynamic temperatures and microwave emissions showed remarkable differences. Thus, contrary to popular opinion, these two processes are not connected. Diseases like stroke and brain degeneration cause increased microwave emissions. Protein denaturation, not thermodynamic temperature, causes this phenomenon. The author’s research may explain the postmortem microwave emission increase. These research findings could inspire new diagnostic methods. Alagee et al. proposed^83^ VIVALDI antenna having a 6.62 dB gain at 2.33 to 7.09 GHz. U-shaped antenna slots boost performance. Etching U-shaped slots improves return loss, bandwidth, and gain by 35.28%, 2.7%, and 10%. The Computer Simulation Technology (CST) package created a four-layer spherical head phantom to test the MWI antenna. The confocal RBMWI is used to reconstruct the image from the scattering parameters collected from the designed antenna for brain stroke detection. Rodr et al.^75^ proposed a compact and low-complexity device for cerebrovascular disease detection and monitoring via microwave technology. The device features a wearable antenna array with twenty-two elements and enables real-time tracking of brain stroke evolution. The imaging algorithm utilizes a differential scheme, reconstructing three-dimensional images using scattering matrices collected at two different time points. The system is validated through numerical and experimental testing, demonstrating accurate localization and monitoring of hemorrhage and ischemia zones with centimetric spatial resolution. Liu et al.^84^ proposed full-waveform autofocus inversion thermoacoustic image reconstruction for non-contact, radiation-free stroke diagnosis. Simulations and measurements update a sound speed distribution-numerical simulation of a human brain model and experimental validation of transcranial thermoacoustic detection in the clinic. Li et al.^85^ proposed orthotopic glioblastoma in vivo could be eradicated precisely and effectively with a pulsed microwave-induced thermoacoustic therapy (MTA). Fedeli et al.^86^ introduce a hybrid MWI method that combines fast qualitative processing with accurate brain dielectric property tomography. This method creates microwave images from simulations and experiments. Three-dimensional, realistic stroke-affected head models and simplified cylindrical phantoms validated the approach numerically and experimentally. The proposed method may lead to a functional clinical imaging prototype in both settings.

#### Breast

Hosseinzadegan et al.^87^ proposed two dimensional (2D) discrete dipole approximation (DDA) for rapid MWT reconstruction. Reconstructing images from synthetic finite element-based solver data and experimental MWI data validates the technique. This method reproduces a 2D cylinder phantom plane in this study. Rebuilding an image takes less than 6 s using the author-proposed forward solver and the nodal adjoint method for calculating the Jacobian matrix. Kaur et al.^88^ proposed a monostatic RBMWI system using a rectangular dielectric resonator antenna (DRA) with a Sierpinski fractal-defected ground structure, which has been simulated and experimentally tested for ultrawideband (UWB) operation to detect breast tumors. High-peak gain fractal DRA covers UWB ranging from 5.6 to 14.2 GHz, having 5.8 dB gain. Breast phantom S-parameter measurements detect tumors. The fractal DRA has rotated 10° in elevation and azimuth in front of the phantom to collect backscattered signals (with/without tumor). Gelatin (skin), petroleum jelly (fat), and wheat flour (tumor) breast phantoms validate S-parameters. MATLAB reconstructs 2D breast tumor images using delay-add-sum with coherence factor and delay-multiply-add-sum with coherence factor from reflection parameters. The breast phantom’s simulated specific absorption rate of the proposed DRA at 6.1 and 13.9 GHz was 1.02 and 1.13 W/Kg, safe for human exposure and efficient with natural dielectric properties. Finally, the monostatic radar-based confocal MWI algorithm located the tumor in the phantom. MATLAB created the tumor image from CST data.

Kaur et al.^89^ proposed a monostatic RBMWI system for breast cancer detection. In RBMWI, the designed DRA is placed parallel to the breast phantom and rotated around it at 10° elevation and azimuthal, first without and then with the tumor. MWI uses 1080 impedance band backscattered signals. The fabricated antenna revolves around an artificial breast that mimics the original properties to verify results. Wheat flour, petroleum jelly (fat), and gelatin simulate breast tissue (tumor). The vector network analyzer (VNA) recorded backscattered signals from the artificial breast phantom with and without tumors at various positions and times. Delay and sum (DAS) and Delay multiply and sum (DMAS) beamforming algorithms processed S-parameters. MATLAB digitizes these signals to detect breast cancer. DMAS detects and characterizes the size of the breast tumor, while DAS detects it. Zhou et al.^90^ proposed studies on ultrasound-guided microwave ablation (MWA) for non-puerperal mastitis (NPM). MWA treatment is effective for NPM with tiny lesions in one quadrant. Larger tumors in two or more quadrants benefited from MWA with incision and drainage. The use of MWA in the study and treatment of NPM shows promising results. Rodriguez et al.^91^ presented a novel MWI device calibration method using real-world measurements and synthetic simulations. The suggested method corrects manufacturing tolerances and positioning-induced antenna array differences from the nominal electromagnetic (EM) scenario. Adult head tissue MWI is measured virtually and experimentally. Full-wave virtual EM analysis software employs the finite element method and accurate three-dimensional computer-aided design models.

### Lungs

Emilov et al.^92^ aims to assess the MWR’s efficacy in identifying pneumonia with complications in individuals afflicted with COVID-19. The MWR showcases an impressive prediction ability, boasting a sensitivity of 98.6% and a specificity of 84.0%. In cases where chest CT may not be accessible or practical, the MWR of the lungs proves to be an effective substitute for diagnosing pneumonia in COVID-19 patients. Table 1 summarizes the state of the art in the application of MWI for disease detection.

**Table 1:** Summary of recent research in MWI domain

Reference	Year	MWI Technique	Methodology	Performance	Target area
79	2022	PMWI	Reflectance measurements were taken using 90 GHz radiometry on two samples.	Radiometric studies show a 0.02–0.27 reflectance difference between healthy and sick skin.	Skin diseases
82	2022	PMWI	Preliminary egg white microwave emission investigations during ethanol-induced denaturation.	Explain postmortem microwave emission increase. This research may inspire new diagnostic methods.	Stroke and brain degeneration
78	2020	PMWI	Placed the MWR sensor on the suprapatellar recess and anterior thigh upper third. Ultrasound and microwave radiometry found fluid and synovitis.	Refining this method, including making sensors for small joints, could lead to the ideal objective tool for clinical, subclinical synovitis detection.	Rheumatoid arthritis and spondyloarthritis
80	2022	MWT	For MWT image reconstruction, the MWI prototype and distorted Born iterative approach algorithms were proposed.	Tracked bone health.	Human calcaneus bone
81	2022	MWT	Proposed a portable electromagnetic tomography brain scanner prototype for clinical trials. The scanner was safe for clinical research.	The scanner was shown to be harmless and functional in both healthy volunteers and actual patients, and it demonstrated early evidence of identifying brain disease in stroke patients.	Brain stroke
77	2021	MWT	Using the MRI reconstruction, develop the human bone representations of different thicknesses.	Provides high-quality images from non-ionizing radiation.	Human bone
87	2021	MWT	2-D discrete dipole approximation for MWT image reconstruction.	The forward solver rebuilds the image in 6 seconds.	Breast Cancer
83	2020	RBMWI	Confocal RBMWI is used to construct the image from the signals collected using the Vivaldi antenna.	The antenna designed for the system gives better results.	Brain stroke
88	2022	RBMWI	UWB breast tumor detection using monostatic was simulated and tested using a rectangle dielectric resonator antenna with fractal-defected ground construction designed by Sierpinski.	1.02 and 1.13 W/Kg, safe for human exposure and efficient with natural dielectric properties.	Breast Cancer
89	2021	RBMWI	MWI uses 1080-impedance-band backscattered signals. The antenna’s artificial breast mimic verifies the results.	MATLAB digitizes signals to detect breast cancer. DAS detects the breast tumor, while DMAS sizes it.	Breast Cancer
84	2022	HBMWI	Full-waveform autofocus inversion thermoacoustic image reconstruction for non-contact radiation.	Simulations and measurements update a sound speed distribution.	Stroke diagnosis
85	2022	HBMWI	Pulsed microwave-induced thermoacoustic (MTA) therapy that could precisely and effectively eradicate.	Microwaves’ deep tissue penetration and ultrasonic shockwave’s rapid decay make MTA therapy for glioblastoma with intact skin and skull promising.	Orthotropic glioblastoma
90	2022	HBMWI	Ultrasound-guided microwave ablation (MWA) research for non-puerperal mastitis.	MWA with incision and drainage helped larger tumors in two or more quadrants. MWA treatment of NPM is promising for research and treatment.	Non-puerperal mastitis
86	2020	HBMWI	A hybrid MWI method combines fast qualitative process with accurate brain dielectric property tomography.	Three-dimensional, realistic stroke-affected head models and simplified cylindrical phantoms validated the approach numerically and experimentally.	Brain
91	2021	HBMWI	Three-dimensional, realistic stroke-affected head models and simplified cylindrical phantoms validated the approach numerically and experimentally.	This method creates microwave images from simulations and experiments. Three-dimensional, realistic stroke-affected head models and simplified cylindrical phantoms validated the approach numerically and experimentally.	Head tissue
75	2023	HBMWI	A compact and low-complexity device for cerebrovascular disease detection and monitoring via microwave technology.	Accurate localization and monitoring of hemorrhage and ischemia zones with centimetric spatial resolution.	Head Imaging

*PMWI* Passive MWI, *MWT* Microwave Tomography, *RBMWI* Radar Based MWI, *HBMWI* Hybrid MWI.

## Artificial intelligence assistance in MWI: techniques and healthcare applications

Microwaves benefit from integrating AI algorithms and techniques as AI enables advanced data processing, image reconstruction, and analysis^93,94^. This integration enhances the accuracy and efficiency of MWI in healthcare applications by leveraging AI^95^. MWI can provide improved diagnostic capabilities and support the development of innovative healthcare solutions^96^.

### AI algorithms

**Fig. 3:**
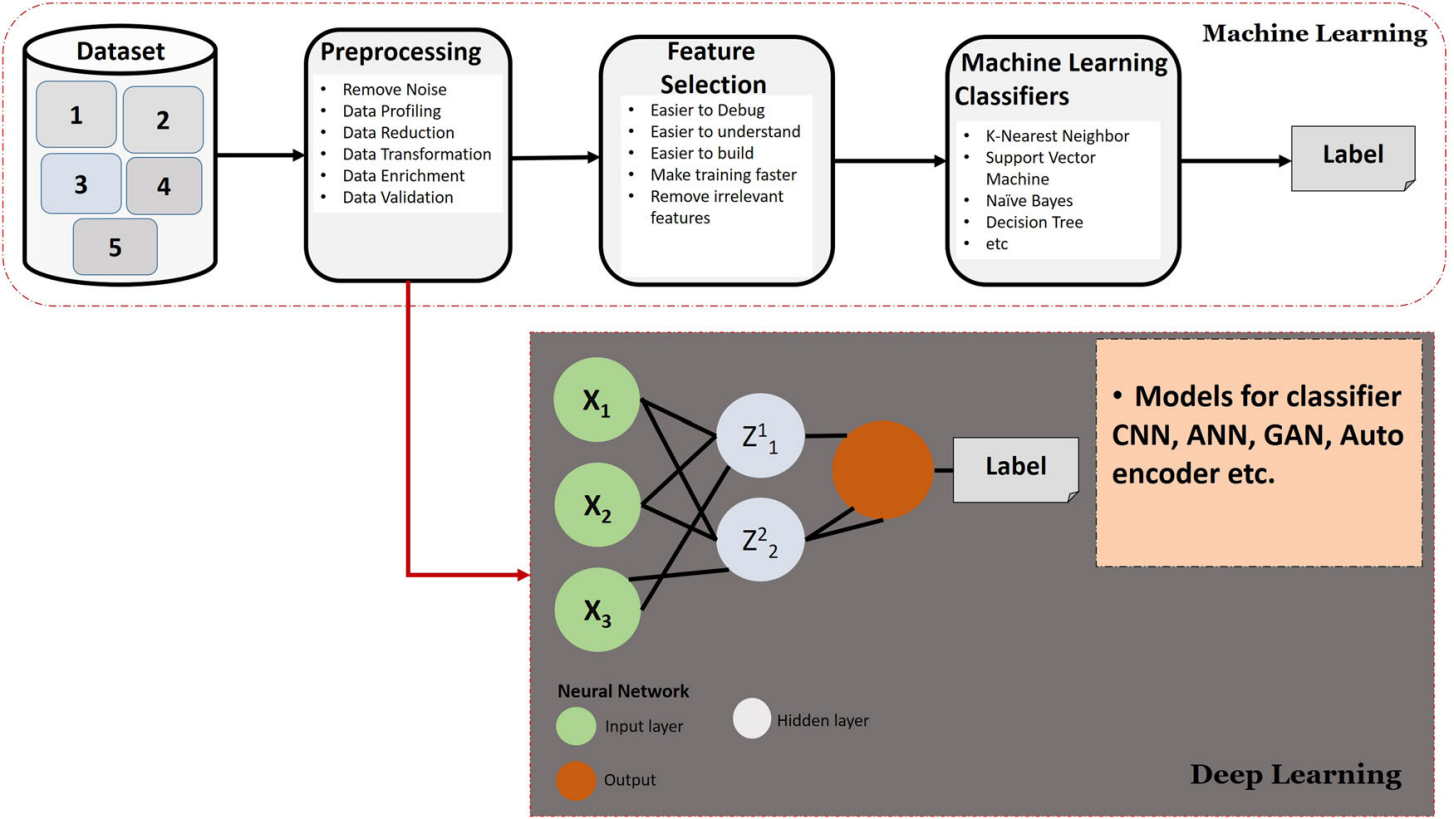
A topology of the general working principle of ML and DL techniques.

ML and DL are two subsets of AI. They employ algorithms to empower computers in learning and making predictions based on data. However, ML and DL possess certain similarities. They also diverge in terms of architecture, complexity, and application. Table 2 shows a summary of some significant works recent in this domain. Figure 3 explains the basic taxonomy of AI algorithms and also details the difference between ML and DL.

**Table 2:** Recent techniques of AI-assisted MWI

Reference	Year	AI technique		MWI technique	Methodology	AI Contribution	Target area
		ML	DL				
146	2023	*✓*	✗	A simplified yet realistic head model consisting of two homogeneous tissues is used in the approach.	A U-Net neural network to predict inner boundaries based on qualitative images obtained using truncated singular value decomposition, the permittivities of the internal domains are iteratively estimated using the distorted Born iterative method.	DL to enhance MWI for estimating tissue permittivities within the head, relying solely on knowledge of the outer head boundary.	Brain stroke
168	2023	✗	*✓*	The study utilized UM-BMID as an open-source experimental database	Objective was to develop an application using MATLAB for breast tumor detection and determination through image processing.	SVM is employed for breast cancer detection using MWI, improving accuracy and reducing false positives and negatives.	Breast cancer
5	2023	*✓*	✗	Proposed a novel approach for solving the inverse scattering problem reliably and automatically.	This approach combines qualitative imaging techniques and DL in a two-step framework.	A neural network retrieves the exact target shape and contrast value by treating the task as image segmentation.	Brain stroke
140	2022	*✓*	✗	A database of numerical breast phantoms has been created using a realistic phantom generator, which accurately simulates various breast tissue properties and structures.	The methodological analysis of the proposed approach focuses on two main areas: the definition and generation of a proper database for neural network training and the design and analysis of different neural network architectures.	The ANN approach was noise-resistant and promising enough to use on realistic anthropomorphic breast phantoms and possibly experimental data.	Breast Cancer
139	2022	*✓*	✗	A labeled synthetic dataset over a range of possible adipose and fibroglandular regions is generated using^169^ approach.	The dataset is collected by replicating the^169^ hardware. Used the data to train neural networks to get prior information about the system like geometry, shape, etc.	Designed neural network not only predicts the geometry and average complex-valued permittivity but also detects the geometry convex hull of the phantom.	Breast Cancer
138	2022	*✓*	✗	Provide solutions to MWI problems. Graph formulation of the MWI array, addressing the challenge of incorporating the physical setup into network structures.	The geometric network GAT uses system topology to process the data most effectively while being lightweight.	A simple model with only 2432 parameters was enough to reconstruct images with acceptable performance.	Breast Cancer
145	2022	*✓*	✗	Constructs a mobile-based MWI head system	The constructed MWHI system captures 400 RMW image samples, encompassing healthy tissue and tumor(s) in various anatomical sites. The YOLO is applied for detection.	The final results show 96% accuracy.	Head Imaging
170	2022	*✓*	✗	Microwave induced thermo-acoustic tomography (TAT) uses circular scanning sensor arrays for signal acquisition.	DL-based method to process time-reversed (TR) TA images. A fully dense U-Net (FD U-Net) is implemented.	Experimental images prove data augmented FDU-Net works.	Medical Imaging
134	2022	*✓*	✗	Microwave-induced thermoacoustic tomography (MITAT) is used for data collection.	Proposes a new DL-enabled MITAT (DL-MITAT) modality for sparse data reconstruction. FPNet + ResU-Net is the domain transform network used. Network design and implementation.	In ex vivo experiments, only 15 measurements can reliably image the breast tumor in full-view and limited-view configurations.	Breast Cancer
143	2022	✗	✗	A technique to join ML with MWI for the classification task focusing. The author solved the data collection challenge by using simulated and measured data.	The algorithm used is MLP for the classification task and for the training of the model used, a linear integral operator that reduces the data generation time concerning the standard full wave.	The activation function is a hyperbolic tangent with the regularization term equal to 0.05. The accuracy reported is 99%, and all the evaluation metrics are close to one.	Brain stroke
133	2022	*✓*	✗	MWR detects breast cancer by measuring tissue thermal properties.	Using the bi-population covariance matrix adaption evolution technique, the weights of the weight-agnostic neural network were optimized (BIPOP-CMA-ES).	Weight-agnostic BIPOP-CMA-ES performed best. It had 163 connections, 0.933 F1-score, 0.932 accuracy, 0.929 precision, and 0.942 recall.	Breast Cancer
132	2022	*✓*	✗	Quantitative MWI ANN approach in real time. It recommends numerical studies to optimize the network design and increase recovery performance and processing time in the MWI framework, an important step toward building future diagnostic applications.	Two key aspects of the suggested methodology are defining and producing a unified database for training neural networks and designing and analyzing various neural network topologies.	Results are promising in qualitative and quantitative comparisons to standard nonlinear inverse scattering techniques.	Breast Cancer
125	2022	✗	*✓*	Models of breast tumors that are anatomically accurate enough for MWI have been developed in preliminary studies.	Preliminary research on three classifiers, LDA, SVM, and KNN, for identifying cancerous and noncancerous tumors.	Compared to the other classifiers, KNN performed best in terms of accuracy (87.5%), sensitivity (83.3%), and specificity (91.7%).	Breast Cancer
129	2021	✗	*✓*	The electric field amplitude of various samples is collected as the experiment data by simulating the human breast in COMSOL Multiphysics.	Microwave signal analysis using SVM is used to differentiate between benign and malignant breast tumors	DT and RF are compared to one another to determine which has superior classification capabilities and reliability.	Breast Cancer
130	2021	✗	*✓*	To predict breast lesions from microwave signals, a quick and precise ML algorithm is proposed.	Raw backscattered signal data is used to train and test an SVM algorithm with a linear and polynomial kernel.	SVM using a third-degree polynomial kernel outperformed state-of-the-art classical ML binary classification techniques, achieving 99.7% accuracy.	Breast Cancer
142	2021	✗	*✓*	The CST simulator models a multilayer head phantom and a 1 cm spherical target representing an intracranial hemorrhage to simulate a circular array-based MIS.	Filtering, edge-detection-based segmentation, K-Means, and fuzzy clustering reveal intracranial hemorrhage areas from reconstructed images.	The proposed method achieves 97% accuracy.	Stroke
124	2021	✗	*✓*	The open-source University of Manitoba-BMI dataset uses breast phantoms in a pre-clinical BMI system (UM-BMID).	Explore the dataset’s usability, implement different ML classification algorithms for tumor detection on UM-BMID, and compare the results to previous publications.	ML in breast MWI has great potential with a maximum accuracy of 94% using RF.	Breast Cancer
135	2021	*✓*	✗	University of Manitoba dataset was used for applying the AI algorithms.	Deep and Convolutional Neural Networks are used to localize, classify, and detect the tumor.	The results outperform existing work on this dataset. The R2 score is 0.33, and F1-score for classification and characterization is more than 0.9.	Breast Cancer
18	2021	*✓*	✗	No major Microwave technique. But important to revolutionize its results.	Used GAN to generate synthetic data similar to MWI to prove the classification efficiency.	The generated data are of high quality and accurately represent the distribution of the original data.	Brain stroke
144	2020	*✓*	✗	MWI 3D images of breast complex-valued permittivity are enhanced using DL.	A U-Net-based 3D Convolutional Neural Network to produce the true 3D permittivity image from CSI 3D images.	The results show that the network performed well with synthetic and experimental data despite being trained only on synthetic data.	Breast cancer
126	2020	✗	*✓*	Utilize signals gathered from a monostatic ultra wideband radar MWI prototype system to categorize breast tumor models in a variety of sizes and forms.	ML algorithms are tailored to detect the tumor. KNN, NB, and DT are used to classify.	On a homogeneous breast phantom, KNN achieved 96.2% classification accuracy, higher than DT and NB ML classifiers.	Breast lesions
141	2020	✗	*✓*	A full head phantom is surrounded by sixteen virtual elements of modified bowtie antennas in a circular array to create an MWI system.	DWT and PCA are two new methods in ML that help with feature extraction and feature reduction. The reconstructed images are then segmented and clustered to look for signs of hemorrhage and stroke using SVM.	The experiment result shows the kernel SVM could classify the 16 images with an accuracy greater than 95%.	Brain stroke

#### ML algorithms

ML algorithms are created to learn patterns and make predictions through training on either labeled or unlabeled data. ML algorithms often employ feature engineering, which involves human experts manually extracting important features from the data to train the model. Common ML algorithms include linear regression, decision trees, support vector machines, and random forests^97^. These algorithms are generally easier to interpret, demand fewer computational resources, and apply to tasks with restricted data or well-defined problem domains. Here are the famous ML algorithms used in MWI.

- K-nearest Neighbour (KNN): The KNN works on the principle that similar data points tend to cluster together; hence items should be in the same class. For the selected value of K, the algorithms find the KNN of the unseen data based on the distance between the existing point and the new one. Distance metrics can be Euclidean, Manhattan, or Minkowski. A similar approach is used for regression despite the class target value being selected^98^.
- K-means Clustering: algorithm performs a series of iterative centroid calculations until the optimal centroid is found. As an outcome, the entire number of clusters has already been determined. Flat clustering is another name for this approach. The term “K-means" describes the maximum number of clusters obtained from a dataset using this method. Then assigning data points to clusters, this method uses a distance measure that minimizes the sum of squared differences between each data point and the cluster centroid. There will be more occurrences of identical data points inside the same cluster if there is less variation between clusters^99^.
- Support Vector Machine (SVM): To classify data points, SVM algorithms typically explore for a hyperplane in N-dimensional space (N being the number of characteristics). The closer data points of a support vector can modify the location and orientation of a hyperplane. These helper vectors maximize the classifier margin. Removing support vectors shifts the plane’s coordinates. These factors underpin SVM^100^.
- Decision Tree (DT): Classification is the most common use of DTs. It’s a tree-shaped classifier with internal nodes for dataset features, branches for decision rules, and leaf nodes for results. Decision trees have a decision and leaf nodes. Decision nodes make decisions, while leaf nodes show the results of previous decisions. Decisions and tests are based on dataset features^101^.
- Random Forest (RF): A random forest algorithm comprises numerous DTs. The ‘forest’ generated by the random forest algorithm is trained using bagging or bootstrap aggregation. The goal of the meta-algorithm known as “bagging" is to increase the accuracy of ML algorithm ensembles. Following the predictions made by the decision trees, the algorithm (random forest) makes a call. It makes predictions by taking the mean of the outputs of multiple trees. As more trees are added, the prediction becomes more precise. A random forest lifts the limitations of a DT algorithm. It improves precision by lowering the propensity to overfit data^102^.
- Extreme Gradient Boosting (XGBoost): In terms of regression, classification, and ranking, this parallel tree-boosting library is unrivaled. Some ML algorithms needed for XGBoost are supervised learning, decision trees, ensemble learning, gradient boosting, etc. Supervised ML can predict unlabeled features in a new dataset using the labels and features used to train a model. By dominating the structured data competitions on Kaggle, XGBoost quickly rose to prominence. These implementations have popularized XGBoost^103^.

#### DL algorithms

DL algorithms, a subfield of ML, draw inspiration from the intricate structure and functioning of the human brain. These algorithms employ artificial neural networks with multiple layers to acquire intricate data representations automatically by doing so. They can learn directly from raw data and remove the necessity for explicit feature engineering within various domains like computer vision, natural language processing, and more. Convolutional neural networks (CNNs) and recurrent neural networks (RNNs) are popular DL architectures. DL algorithms excel in tasks demanding extensive data processing and have achieved impressive state-of-the-art outcomes in areas such as image and speech recognition, natural language understanding, and generative modeling. However, it is important to note that DL models often require significant computational resources and vast amounts of labeled data to train effectively^104^.

- Artificial Neural Network (ANN): These networks mimic their biological counterparts by borrowing critical concepts from human neural systems. An ANN model’s specific function is to simulate the brain’s and nervous system’s electrical activity. An ANN’s node layers include an input layer, a hidden layer(s), and an output layer(s). Each “node" is an artificial neuron that interacts with one another via weight and threshold linkages. If a node’s output exceeds the set threshold, it becomes active and passes its data on to the next network layer. If this is not the case, no information is passed to the lower layer of the network^105^.
- Convolutional Neural Networks (CNN): In AI, a CNN is a network optimized for processing data with a grid-like topology, such as an image. Visual information is encoded in a binary format known as a digital image. There are several squares, or pixels, laid out in a grid, and those squares have numbers associated with them that indicate the intensity and color of the pixels they represent^106^.
- Autoencoders: are feedforward neural network subtypes with identical input and output. They reduce the number of dimensions of the input and use that representation to reconstruct the output. Latent-space representation is a compressed version of the input^107^.
- Generative Adversarial Neural Network (GAN): Two NNs in a DL model, a GAN, use a competitive learning process to improve their predictive abilities. GANs typically operate unsupervised in their learning process and employ a cooperative zero-sum game structure. GANs are composed of two neural networks, the generator, and the discriminator. The discriminator and the generator both use NNs, but the generator uses a deconvolutional NN, while the discriminator uses a convolutional NN. The outputs produced by the generator are meant to be so realistic that they may be mistaken for actual data. The discriminator’s job is to determine which inputs it receives manufactured by the generator. In a nutshell, GANs generate their datasets to learn from. The generator will start producing better-quality output, and the discriminator will get better at identifying falsely generated data, so long as the feedback loop between the adversarial networks is running^108^.
- Graph Attention Network (GAT): To overcome the limitations of current systems based on graph convolutions or their approximations, GAT proposes a novel NN architecture that can operate on graph-structured data. By stacking layers in which nodes can attend over the characteristics of their neighborhoods, a GAT enables implicitly identifying distinct weights for various nodes in a neighborhood without requiring any costly matrix operation (such as inversion) or having to rely on prior knowledge of the graph structure^109^.

### History of AI-assisted MWI in clinical setting and research

Wang et al.^110^ presented a neural network-based approach for MWI in medical diagnosis for the first time. The main objective is to reconstruct the complex permittivity of biological tissues illuminated by transverse magnetic (TM) incident waves. To address the challenge of the ill-posed nature of the inverse scattering problem, we introduce a stochastic process based on Markov random field and prior knowledge. Our proposed solution involves a coupled gradient neural network that can effectively handle the mixed-variable problem. The reconstructed dielectric permittivities are continuous complex variables, while the line processes preserve the reconstructed image’s edges are binary variables. Rekanos et al.^19^ proposed using radial basis function neural networks (RBFNNs) in the inverse-scattering problem for microwave medical imaging. The aim is to estimate tissues’ geometric and electromagnetic properties by analyzing scattered-field measurements obtained during electromagnetic wave illumination of the body. The RBFNNs are trained using the orthogonal least-squares algorithm, allowing for straightforward network construction and determination of free parameter values. The methodology is applied to detect and monitor leukemia by estimating the position and size of proliferated marrow within a bone. Kerhet et al.^111^ proposed a 3-D approach based on an SVM classifier to detect tumor locations using confocal MWI. Instead of solving the computationally intensive inverse scattering problem, the SVM classifier transforms its output into a posteriori probability of tumor presence. Microwave data, including noisy environments, are generated using the finite element method with impedance boundary conditions. The resulting probability maps effectively highlight the region surrounding the tumor location, distinguishing it from the background of overall probability values.

Woten et al.^112^ explored using micromachined antennas operating at microwave frequencies and an ANN detection tool for preprocessing tumor detection. The antennas were designed to be compact, enabling an array to be placed around the breast to concentrate the beam on the tumor. The ANN offered a statistical assessment of tumor presence by utilizing scattered fields from the tumor. The network’s accuracy relied heavily on the training procedure investigated in this research. Abbosh et al.^113^ employed a feed-forward back-propagation neural network to detect and locate early breast cancer using scattered signals from a three-dimensional breast model. The proposed method achieves promising results, with 100% success in tumor detection and 95% success in localization using the neural networks and electromagnetic simulator. Yahya et al.^114^ proposed a combination of wavelet transform and neural networks investigated for early breast cancer detection and diagnosis. Using a three-dimensional breast model, the proposed algorithm achieves promising results, with 100% success in tumor detection. The method also demonstrates a high correct recognition rate for tumor size, ranging from 65.52% to 100%, depending on the tumor radius. Hahn et al.^115^ a systematic approach is introduced for designing phantoms that closely resemble breast tissues in terms of dielectric properties and realistic physical models. The approach utilizes a regression model to match the dielectric properties of the phantoms with those of breast tissues. This method can be applied to create phantoms for various tissue types using measured dielectric properties, enabling the development of accurate benchmarking phantoms for testing MWI algorithms.

**Fig. 4:**
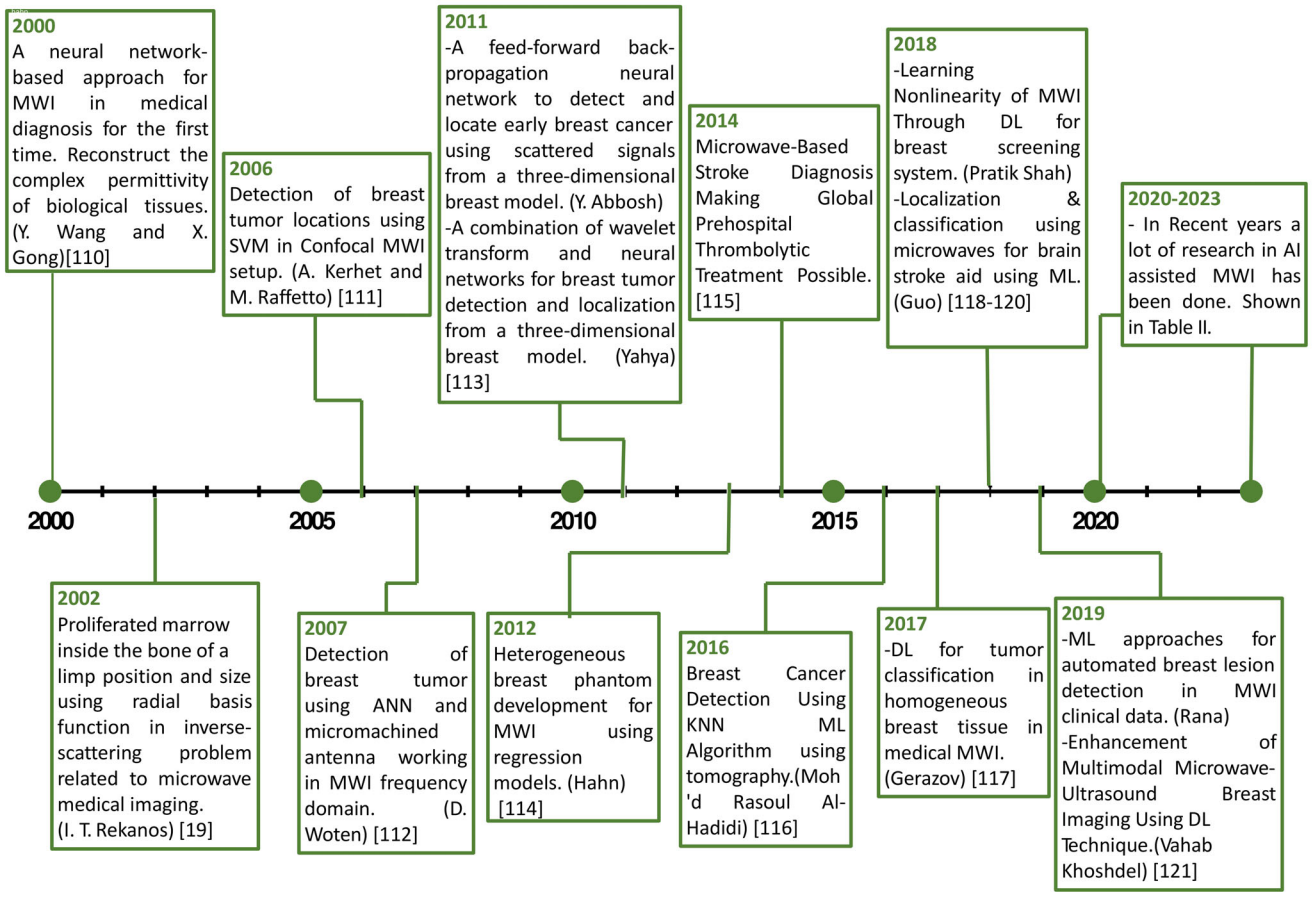
Evolution of AI-assisted MWI in biomedical applications.

Persson et al.^116^ utilized a head-worn antenna system to perform microwave scattering measurements. The clinical objective of the project was to enable early diagnosis and treatment of ischemic stroke patients, as microwave systems have the potential for prehospital use, allowing for faster intervention compared to current methods. The method involves two main parts: image processing techniques are applied to prepare mammography images for feature and pattern extraction, followed by the utilization of the extracted features as inputs for supervised learning models such as Back Propagation Neural Network (BPNN) and Logistic Regression (LR)^117^. Gerazov et al.^118^ proposed that DL on a small dataset can improve tumor classification in breast MWI simulations. The hybrid system achieved the best results when DL feature embedding was combined with a standard SVM classifier. The dataset was obtained from numerical simulations of tumor models embedded in homogenous breast adipose tissue in the Finite Difference Time Domain (FDTD). The accuracy obtained is 93.44%, which outperforms already existing conventional ML techniques. Guo et al.^119^ offered a system for localization and classification using microwaves for brain stroke aid. The MWI technique implemented is MWT, and the ML algorithms that aid the system are k-means clustering and SVM. The Born iterative approach feeds k-means clustering, used for feature extraction of the brain’s dielectric profile’s amplitude. Clusters are feature vectors for SVM creation and evaluation. For classification, MRI-derived realistic head phantoms with varying signal-to-noise ratios are employed. The receiver operating characteristic curve (ROC) is used to evaluate the efficiency of the proposed framework. 2D categorization results show 91% sensitivity and 87% specificity. A DL approach is used to accurately estimate the total electric field by leveraging a Born-approximated solution. The learned field is further utilized to estimate the permittivity and conductivity, demonstrating the method’s effectiveness in a 2D breast MWI problem^120^. Rana et al.^121^ first use of the KNN classifier to detect breast cancer in MWI data. The classifier predicts using only hyperspace feature proximity without assuming anything about the data distribution. The author compared neighboring feature vectors using Euclidean distance. K = 1, which uses 40% of training data, outperforms other NNs. Testing showed 0.608 (60.8%) accuracy and 0.541 (54.1%) sensitivity. It correctly identified lesions in 66.77% of patients by using an SVM to develop a smart classification system for helping doctors spot breast lesions using the MWI system. Statistical measures and careful analysis revealed that the breast data might be classified using the quadratic kernel of SVM with a high degree of accuracy of 98%. Figure 4 shows the taxonomy of AI-assisted MWI in healthcare

### State-of-Art techniques of AI-assisted MWI in disease diagnosis

#### Phlebology

Levsinskii et al.^122^ proposed a novel AI model, using MWR data, has been developed for predicting the disease status of phlebology patients. The model utilizes both MWR and infrared (skin temperature) data from the lower extremities to create a feature space and build a classification algorithm. This approach achieves a sensitivity exceeding 0.8 and a specificity surpassing 0.7. Additionally, the model delivers advisory results presented in a manner comprehensible to clinicians. The author’s objective of this research is to propose a model that dynamically characterizes the condition of patients with venous system diseases based on MWR and Infrared (IR) data. This initiative seeks to establish the groundwork for an AI diagnostic system mechanism.

#### Bones

Beyraghi et al.^123^ investigate the viability of employing a deep neural network (DNN) for diagnosing bone fractures by utilizing non-invasive radio frequency wave propagation. In contrast to previous methods relying on X-ray images, this approach involves training the DNN with S-parameters profiles to circumvent labeling and data collection challenges. This developed network can concurrently categorize various intricate fracture types (normal, transverse, oblique, and comminuted) while estimating crack lengths. This system holds potential as a portable device for swift preliminary diagnosis in emergency scenarios, such as ambulances, retirement houses, and low-income settings where expert radiologists may not be readily available. The datasets are generated by accurately modeling the human body and adjusting tissue diameters to simulate diverse anatomical regions. Numerical results indicate the successful training of the DNN without overfitting. To validate these findings, the author conducted experiments on sheep femur bones covered by a liquid phantom, revealing that fracture types can be accurately classified without resorting to potentially harmful and ionizing X-rays. AI-aided diagnostic bone fracture detection system helps to gain autonomy of the system by using DNN for classification and length estimation of the bone crack without the need for an expert radiologist. Moreover, the system’s portability makes it suitable for remote areas and the problem of the dataset collection and labeling will be easily resolved using an AI-based system.

#### Breast

Patel et al.^124^ conducted research to explore the working efficiency of the breast cancer detection dataset collected by the University of Manitoba using ML algorithms. Different experimental conditions and data manipulations have resulted in multiple dataset versions namely generation one and generation two. The author uses a pre-processed version of this dataset to train high-quality ML models, alleviating some of the models’ computational burdens. Once a dataset has been assembled, it is immediately put through a train-test split so separate data sets can be used for training and testing models. This method was applied to testing multiple models, and the important metrics for comparing them were extracted. Maximum accuracy for DT was 84%. Using random forest, the author obtained 94% accuracy. Obtained 84% using XGBoost on the Breast Cancer dataset using MWI setup for data collection. The results show the promising contribution of ML in the detection of breast cancer.

Peli et al.^125^ classify tumors in magnetic resonance images as malignant or benign based on their morphological features; a preliminary investigation was conducted using three classifiers where KNN outperforms with the accuracy of 85% than linear discriminant analysis (LDA) and support vector machine (SVM).

Conceiccao et al.^126^ proposed a monostatic MWI prototype system for tumor-filled breast phantoms at 1–6 GHz. These tumor replicas were implanted into one of two breast phantoms, one with a uniform cellular structure for breast homogeneity and the other with groups of fibro glandular resembling tissue properties for heterogeneity. Tuned Naive Bayes (NB)^127^, Decision Trees (DT)^101^, Principal Component Analysis (PCA)^128^, and KNN classifiers were used to classify cancerous cells from healthy one in the breast phantoms. KNN classified tumor models in breast phantoms with a 96.2% accuracy in the classification task. The KNN algorithm outperformed the DT and NB classifiers in global classification.

Chen et al.^129^ demonstrate that dividing breast tumors into benign and malignant types based on their dielectric properties is a viable option. By modeling the human breast in COMSOL Multiphysics, the author gets experimental data by measuring the electric field amplitude of several samples. Microwave signal analysis using SVM differentiates between benign and malignant breast cancers. Sami et al.^130^ implemented both linear and polynomial kernels to evaluate SVM’s efficacy. The model is examined using feature extraction and dimensionality reduction. For dimensionality reduction, the author used PCA. On MWI’s dataset, SVM’s specificity and sensitivity were assessed. Testing data was 94% accurate. Polynomial kernels used second- and third-degree polynomials. SVM training used 75% of the data. SVMs with polynomial kernels have 99.7% accuracy on K-fold cross-validation^131^.

Ambrosan et al.^132^ proposed an ANN real-time quantitative MWI strategy. The author suggests improving recovery performance and time processing using numerical studies by optimizing neural networks focusing on MWI in breast analysis. This is an essential step in future diagnostic applications. The two main steps are defining and creating a neural network training database and designing and analyzing neural network topologies. The approach was evaluated in numerically noisy settings with varying SNR values, demonstrating its robustness against noise. The outcomes of qualitative and quantitative comparisons to standard nonlinear inverse scattering techniques are extremely encouraging. Employing quantitative MWI and NN can be a viable alternative to traditional medical imaging procedures because they are less expensive, safer, faster, and quantitative, and ideal for assisting medical decision-making.

Li et al.^133^ explored new ways to automate the search for optimal neural architecture to boost classification performance. After determining the network architecture, the author explored improving the weights of the weight-agnostic neural network with the bi-population covariance matrix adaptation evolution strategy (BIPOP-CMA-ES). Results: The experimental subjects were 4912 patients with known breast cancer risk profiles. The best overall performance was found in the weight-neutral BIPOP-CMA-ES model. It achieved an F1 score of 0.933, 97.32% accuracy, 97.29% precision, 94.42% recall, and 163 associations. The model’s results show promise for MWR as a diagnostic tool for cancer diagnosis based on neural networks. The total performance can be enhanced by decoupling the topology search from weight training.

Zhang et al.^134^ for sparse data reconstruction and breast cancer detection proposes DL-enabled microwave-induced thermo-acoustic tomography (DL-MITAT). FPNet+ResU-Net is the network for transforming domains. Explanation of the design and implementation of the network. The DL-MITAT method is evaluated via computer modeling and ex vivo breast phantom experiments. The trained network outperforms conventional imaging algorithms by using fewer artifacts and producing higher-quality images. A breast tumor image can be reliably recovered from 15 measurements in ex vivo experiments.

Alkhatib et al.^135^ proposed DL frameworks comprised of convolutional layers in deep NNs (DNN) to aid tumor detection, localization, and characterization using scattering parameters and metadata characteristics. Compared to existing methods in the literature, the created DL framework provides state-of-the-art tumor detection, localization, and characterization results. These encouraging findings show the possibility and advantages of using DNNs trained on real-world scattering parameter observations to perform breast MWI.

Shao et al.^18^ proposed a neural network that can translate measured microwave signals received from a 24 × 24 antenna array operating at 4 GHz into an image of 128 × 128 pixels. To lessen the difficulty of training, the author first created an autoencoder that represented high-resolution images 128 × 128 as 256 × 1 vectors; then created a second neural network that mapped microwave signals to the compressed features 256 × 1 vectors. When both NNs are effectively built, they can be joined to form a complete network for reconstruction. The two-stage training technique makes it easier to train DL networks (DLNs) for inverse reconstruction. Simulated instances and experimental data verify the proposed NN, including objects of various forms and sizes, locations, and dielectric constants ranging from 2 to 6.

Ekblom et al.^136^ aim to investigate the feasibility of using a GAN to generate microwave data to supplement the current dataset and improve the effectiveness of a stroke detection algorithm. The research’s main difficulties stem from the limited data set size and the complex nature of the samples. To generate the data, a Conditional Wasserstein GAN was used. The author also decided to look into the effects of including DeLiGAN^137^ because of the low data regime. The methods for assessing GAN-generated data are also discussed in this paper. A separate classifier network is used to assess the generated data’s accuracy. Classification problem evaluations and distribution coverage visualizations show that the generated data are of high quality and accurately represent the distribution of the original data. Nonetheless, the findings also demonstrate that the generated data is not a perfect substitute for the real data and is judged to be lacking in some quality measures. Nevertheless, the paper results are encouraging, and it is concluded that it is possible to generate microwave data that will be used for stroke detection, with great scope for further improvements.

AlSaffar et al.^138^ proposed a graph model formulation solution to the MWI problem. The geometric network uses system topology to process the data most effectively while being lightweight. It was discovered that a basic model with only 2432 parameters was sufficient to reconstruct images with satisfactory performance. Because node-level computation only generates partial solutions, the model cannot see the final solution until it is aggregated. The model’s ignorance of the overall answer was advantageous in several ways, including that it automatically allows the model to generalize since the sum of any number of partial solutions can equal any number of total solutions. For the same reason, it inevitably implies that the model is immune to overfitting.

Edward et al.^139^ demonstrated the impressive ability of a neural network, trained using synthetic data, to analyze experimental data through parametric inversion accurately. This neural network effectively extracted relevant prior knowledge, such as the geometry and average complex-valued permittivity, which are crucial for understanding the fibroglandular tissue in a simplified model of the human breast. The neural network can also detect the geometry of the convex hull of the phantom. Ambrosanio et al.^132^ proposed an ANN approach for efficient and real-time quantitative microwave breast imaging. The work includes numerical analyses to optimize the network architecture, enhance recovery performance, and reduce processing time within the microwave breast imaging framework.

Albaaj et al.^140^ objective was to develop an application using MATLAB for breast tumor detection and determination through image processing. The study utilized UM-BMID as an open-source experimental database. The paper focuses on template design, algorithm formation, and key features such as image acquisition, noise reduction, tumor region identification, and marking. A Graphical User Interface (GUI) is incorporated for user-friendly interaction, providing information on tumor severity and necessary steps for treatment. SVM is employed for breast cancer detection using MWI, improving accuracy and reducing false positives and negatives.

#### Brain

Ojaroudi et al.^141^ propose a unique ML-based post-processing method for detecting brain hemorrhage stroke utilizing MWI equipment. MWI uses a circular array of sixteen modified bowtie antennas around a head phantom in CST medium-a novel ML method using the feature extracting discrete wavelet transform and reduction inducing PCA. After SVM segmentation and clustering, a reconstructed image is used to identify bleeding strokes. The simulations showed the method could accurately locate and categorize bleeding targets.

Roohi et al.^142^ proposed an MWI-based method for detecting and classifying intracranial hemorrhage strokes using ML techniques. Sixteen modified bow-tie antenna elements are arranged in a circle around a multilayer head phantom serving as an intracranial hemorrhage target. The system is modeled in the CST simulator. K-means and fuzzy clustering, edge-detection-based segmentation, and filtering algorithms are just some of the new compound KNN approaches used to discern between healthy and diseased brain structures from rebuilt images.

Mariano et al.^143^ proposed a technique to join ML with MWI for the classification task focussing on brain stroke application. The author solved the data collection challenge by using simulated and measured data. The algorithm used is MLP for the classification task. He used a linear integral operator for the model’s training that reduced the data generation time concerning the standard full wave simulation. The dataset consists of nine classes based on the kind and location of stroke in the brain. The total samples are 4500, and the dataset is divided into 80% for training and 20% for testing of the MLP. At the same time, the MLP is designed so that it has four layers having 1000, 500, 250, and 100 neurons, respectively.

Khoshdel et al.^144^ proposed a 3D CNN U-Net trained with images obtained via Contrast-Source Inversion (CSI) to reconstruct the real 3D permittivity. To reconstruct CSI, the author synthesized the scattered microwave field using 3D CSI images and true numerical phantom images. Hossain et al.^145^, introducing a DL-based YOLOV5 object detection model in the mobile head imaging system (MWHI) for automatically classifying and detecting human brain disorders. The models are trained with 80% of the images and then tested with 20%. Later, 20% the dataset is used to validate the models once they’ve been trained on the remaining 80%. The detection and classification outcomes are evaluated utilizing the YOLOv5 model in its three versions: YOLOv5s, YOLOv5m, and YOLOv5l. The YOLOv5l model is more effective than its forerunners, YOLOv5s and YOLOv5m, and the state-of-the-art object identification algorithms available today. For the YOLOv5l model, the final results were 96.32% accuracy.

Yago et al.^5^ proposed a novel approach for solving the inverse scattering problem reliably and automatically. This approach combines qualitative imaging techniques and DL in a two-step framework. In the first step, the orthogonality sampling method processes scattered field measurements into an image, providing an estimate of target shapes and encoding information on their contrast values. In the second step, a neural network retrieves the exact target shape and contrast value by treating the task as image segmentation. The approach is validated using synthetic and experimental data, including comparisons with existing literature, and its potential for biomedical imaging is demonstrated through a numerical example in microwave brain stroke diagnosis.

Ninković et al.^146^ a DL enhanced MWI approach for permittivity estimation of head tissues in brain stroke diagnostics. The proposed method leverages a U-Net neural network to predict inner boundaries based on qualitative images obtained using truncated singular value decomposition. Subsequently, the permittivities of the internal domains are iteratively estimated using the distorted Born iterative method. The approach is evaluated using a simplified but realistic head model comprising two homogeneous tissues, demonstrating its potential for accurate permittivity reconstruction in MWI applications.

### Benefits of AI-assisted MWI systems

The recent literature review on the integration of Artificial Intelligence (AI) into Microwave Imaging (MWI) systems underscores several potential advantages. Machine Learning (ML) and Deep Learning (DL) techniques enable AI to significantly enhance diagnostic accuracy by discerning subtle patterns and anomalies in MWI data, which may pose challenges for human interpretation^95^. Moreover, AI contributes to the automation of repetitive tasks within MWI analysis, alleviating the burden on healthcare professionals and potentially expediting the diagnostic process. Additionally, AI-assisted MWI systems exhibit the capacity to improve both sensitivity and specificity in disease detection, achieving a balance that minimizes false positives and false negatives^122^. Furthermore, the continuous learning and adaptation capabilities of AI models, informed by new data, result in improved performance over time and ensure alignment with evolving understandings of MWI patterns and diagnostic criteria.

## Insights and pitfalls

Recent research advances in AI-assisted MWI systems have shown promising results, paving the way for developing clinically intelligent technologies with user-friendly features. However, several limitations must be overcome before creating a sophisticated, seamlessly integrated solution miming human capabilities.

### Limited training data

AI models need a significant amount of high-quality training data to learn patterns and generate accurate predictions effectively^147^. A significant constraint in AI lies in its capacity to make decisions when faced with incomplete or restricted information. Despite the capability of AI algorithms to scrutinize extensive data sets and recognize patterns, they cannot comprehend context and formulate decisions guided by intuition or common sense. Consequently, in scenarios marked by ambiguity or uncertainty, AI may struggle to arrive at optimal decisions. Furthermore, the learning process of AI systems is contingent upon the data they are exposed to. Consequently, if the data harbors biases or exhibits gaps, the AI system might generate decisions influenced by these biases, potentially resulting in inaccurate or unjust outcomes. Nevertheless, it can be quite challenging to acquire diverse and annotated MWI datasets as only limited open-source MWI datasets are present. Researchers are currently dedicated to constructing comprehensive and explainable curated datasets to enhance the performance and generalizability of AI models in the field of MWI^148^.

### Generalization to different conditions

AI models dedicated to specific MWI datasets may struggle to achieve robust generalization across a wide range of imaging situations, patient groups, or hardware differences. The complexities of medical imaging include a wide range of variables, such as varied imaging methods, patient demographics, and various device configurations. As a result, the nuances contained in the training data may not fully represent the range of probable events experienced in real-world medical applications. As a result, when confronted with unexpected conditions or populations that were not well represented during the training phase, the flexibility and generalizability of such AI models may be jeopardized. This constraint highlights the importance of tactics that increase the inclusion and diversity of training datasets, allowing for the development of AI models capable of performing increasingly complex tasks^149^.

### Interpretability and transparency

The lack of interpretability and transparency in AI models can be a limitation in healthcare settings. In healthcare, it’s a big challenge when AI models operate like mysterious “black boxes." This means their decision-making is unclear, making it tough for healthcare professionals to grasp the reasoning behind specific suggestions or predictions. This lack of transparency makes it hard to build trust in AI systems, which is crucial for seamlessly integrating them into healthcare practices. In the medical field, where decisions directly impact patients, understanding AI outputs is essential. Clear and transparent AI models not only foster better teamwork between healthcare providers and AI but also help identify and address potential biases or errors, ensuring the safe and effective use of AI in healthcare^150^.

### Real-time processing

The imperative for real-time processing in clinical settings underscores a key challenge in the application of AI-assisted MWI, particularly when employing DL models. While DL models exhibit remarkable capabilities in discerning intricate patterns and generating insightful predictions from voluminous data, their computational intensity can result in prolonged processing times. The developed network architectures and the computations involved in DL can demand substantial computational resources, potentially impeding the swift processing essential in clinical environments where timely decisions are critical. Balancing the formidable computational demands of DL models with the imperative for real-time applications becomes a pivotal consideration in harnessing the full potential of AI-assisted MWI within clinical settings. Striking this balance requires innovations in hardware capabilities, optimization techniques, or the exploration of alternative model architectures to ensure that the computational intensity inherent in DL does not compromise the timely delivery of insights in healthcare scenarios^151^.

### Ethical considerations

The integration of AI technology introduces a myriad of ethical considerations, prompting scrutiny and deliberation. Among the foremost concerns are issues related to privacy, data security, and bias, which collectively shape the ethical dimensions of AI in healthcare. The utilization of AI in healthcare often involves the processing and analysis of sensitive patient data, raising profound questions about the protection of individual privacy. As AI systems draw insights from vast datasets containing personal health information, there is a compelling need to establish robust frameworks that ensure the confidentiality and protection of patient data. Data security emerges as another critical ethical facet, as the susceptibility of healthcare systems to cyber threats poses a potential risk to the integrity and accessibility of patient information. Security against unauthorized access and breaches becomes imperative to maintain the trust and integrity of healthcare AI applications^152^.

### Validation and regulatory approval

Clinical practice mandates a meticulous process of validation and regulatory approval, emphasizing the imperative of robust clinical trials. The essential undertaking of clinical trials is integral to comprehensively assess the performance of AI models, substantiate their safety, gauge their efficacy, and ascertain their overall reliability in healthcare applications. These trials serve as a pivotal mechanism to validate the clinical utility and suitability of AI-assisted MWI, providing empirical evidence that informs regulatory decision-making. Rigorous evaluation not only ensures the alignment of AI models with established standards of medical practice but also patient well-being by affirming the accuracy and dependability of these technologies. Regulatory approval processes, often overseen by health authorities, play a crucial role in validating the scientific merit and clinical validity of AI applications, instilling confidence in healthcare professionals, and the responsible deployment of AI-assisted MWI in diverse clinical settings. Consequently, the convergence of systematic clinical trials and regulatory scrutiny establishes a foundation for the ethical, safe, and effective integration of AI technologies, reaffirming their credibility and utility within the rigorous framework of clinical practice^153^.

### Integration with existing workflows

The seamless integration of AI-assisted MWI into extant healthcare workflows emerges as a pivotal determinant for its practical adoption. This underscores the necessity for the compactenss of AI technologies within the established structures and processes of healthcare delivery. Practical adoption hinges upon the ability of AI-assisted MWI to align with and enhance existing workflows rather than imposing disruptive alterations. Achieving this cohesion demands a nuanced understanding of healthcare processes, workflow dynamics, and the specific needs of healthcare professionals. Moreover, it underscores the importance of developing AI applications that are intuitive, interoperable, and capable of seamlessly interfacing with prevalent healthcare systems. The practical adoption of AI-assisted MWI further necessitates considerations of user experience, addressing the usability concerns of healthcare practitioners, and minimizing potential disruptions to clinical routines. Human-centric design principles play a crucial role in this context, ensuring that AI technologies become supportive tools rather than sources of additional complexity^154^.

## Future directions

**Fig. 5:**
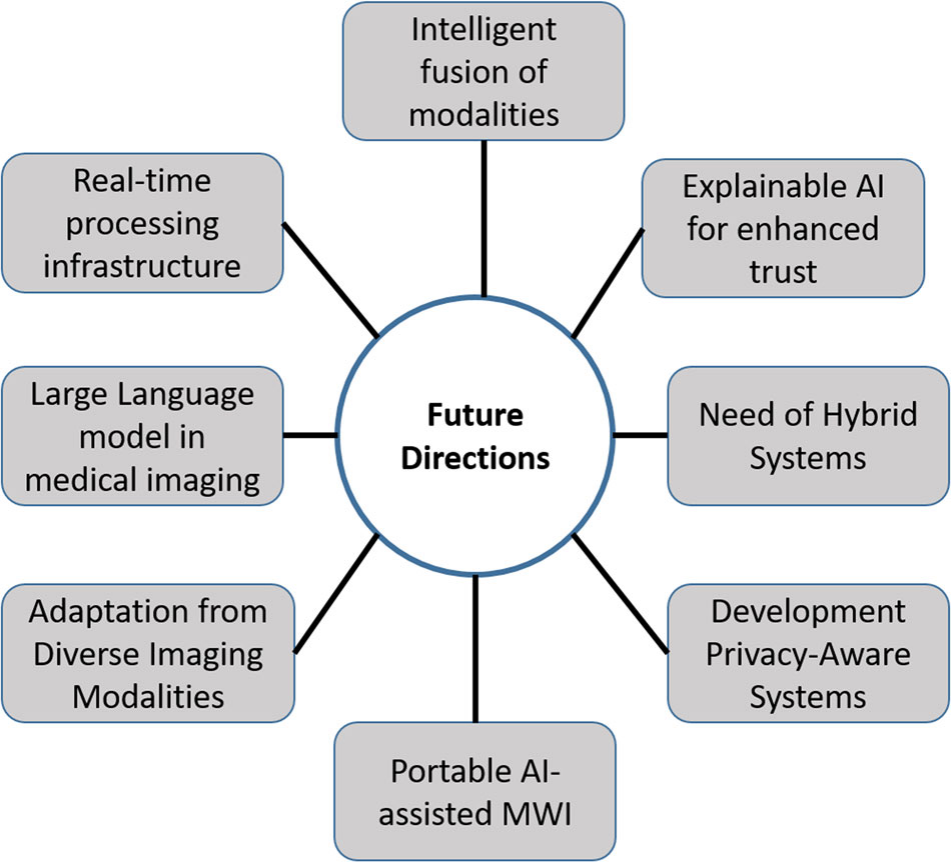
Future directions for AI-assisted MWI efficient development.

The future directions are discussed here in detail. Figure 5 shows the future directions for AI-assisted MWI efficient development.

### Intelligent fusion of modalities

Researchers are working on the fusion of different medical imaging modalities and has been successful^155^. Similarly, AI-assisted MWI integrates with other imaging modalities, such as MRI, ultrasound, or X-ray, by intelligently fusing complementary information from multiple modalities^156^. In that case, clinicians can benefit from a comprehensive and synergistic imaging approach, enhancing diagnostic accuracy and enabling personalized treatment planning.

### Real-time processing infrastructure

TinyML has the potential to significantly impact the development of a real-time processing infrastructure for AI-assisted MWI (MWI), by optimizing and compressing deep learning models. TinyML enables on-device analysis of MWI data. This eliminates the requirement for extensive computational resources and reduces latency. As a result, actionable insights can be generated immediately at the point of care enabling on-the-spot diagnosis, treatment guidance, and surgical interventions. The compact and power-efficient nature of TinyML allows for seamless and efficient delivery of real-time decision support in AI-assisted MWI^157^.

### Large language model in medical imaging

In medical imaging, large language models (LLMs) offer valuable contributions, particularly in image captioning and radiology reporting. These models excel at generating descriptive and contextually relevant text based on medical images, aiding healthcare professionals in the interpretation and communication of intricate diagnostic information. This not only enhances the efficiency of radiologists but also improves the clarity of reports for referring physicians. Additionally, LLMs play a crucial role in supporting the creation of comprehensive medical knowledge bases, providing quick access to pertinent information for healthcare practitioners. Their aptitude for understanding and generating text facilitates the extraction and summarization of medical literature, ensuring that healthcare professionals stay abreast of the latest research and developments. While LLMs may not directly analyze medical images, their integration into healthcare systems enhances natural language interfaces, automates documentation processes, and elevates overall communication and information accessibility in medical modalities^158^.

### Adaptation from diverse imaging modalities

By analyzing how AI is effectively utilized in diverse imaging modalities such as radiology, pathology, and diagnostic imaging, we aim to extract valuable insights that can inform and potentially enhance the development of AI-assisted MWI. Understanding the methodologies and successes in these analogous imaging domains provides a foundation for identifying commonalities, addressing challenges, and discerning best practices. For instance, the deployment of AI in radiology for tasks like image segmentation, detection, and classification has demonstrated noteworthy advancements^159^. Analyzing these applications offers a wealth of knowledge that can be applied to optimize the adaptability, performance, and efficacy of AI algorithms in the unique context of MWI. The integration of AI holds immense potential for advancing MWI across multiple dimensions. AI algorithms can substantially enhance the signal-to-noise ratio (SNR) and contrast-to-noise ratio (CNR), contributing to heightened image quality in MWI as they do in the case of CT angiography^160^ and PET^161^. Furthermore, AI-driven feature extraction techniques can refine the identification of relevant anatomical and pathological structures, fostering more accurate diagnostic information. Predicting gold standard parameters becomes more robust through AI, offering clinicians valuable insights for precise medical assessments as it does for other modalities^162^. Super-resolution and image reconstruction benefit from AI algorithms, enabling the generation of high-resolution images with enhanced clarity. In terms of speed and dynamics, AI optimization facilitates real-time data processing and can elevate MWI’s applicability in dynamic clinical environments like in next-generation healthcare and biomedical platforms^163^. Intelligent algorithms can also contribute to hardware improvements, making efficient use of existing resources and potentially obviating the need for costly upgrades. AI-driven personalization, particularly through prognosis prediction, empowers tailored healthcare solutions by considering individual patient characteristics^164^, thereby promising a transformative impact on the future landscape of MWI applications.

### Explainable AI for enhanced trust

Ensuring the successful integration of AI-assisted MWI systems within clinical settings is necessitated. A comprehensible AI framework that instills confidence among doctors and patients alike. The solution lies in harnessing explainable AI technology, which empowers AI models to provide insight into their purpose, intended consequences, and inherent biases. Decision aids driven by AI facilitate the assessment of model precision, fairness, transparency, and desired outcomes. For an organization to gain trust and confidence in putting AI models into production, explainable AI is necessary^165^.

### Need of hybrid systems

As was previously mentioned, the researchers are attempting to develop hybrid systems. The hybrid technique helps MWI by employing several different methods to address the method’s shortcomings. Hybrid systems allow for enhanced performance in clinical settings like superior diagnostic accuracy through the integration of multiple technologies, enabling personalized treatment and real-time monitoring for optimized patient care. The researchers are currently working to make even more progress in this area

### Portable AI-assisted MWI

Given the advantages of mobile imaging equipment for quality care and clinical readiness in natural catastrophes, creating portable MWI devices is an emerging field^166^. Portal AI-assisted systems can reduce the number of modalities per year. However, for implementing AI models on portal devices, the need for tinyML came so that a compressed-size model can work fine on a small setup.

### Development privacy-aware systems

Data privacy and security have become central concerns in modern life. They deal with people’s private information rights, especially where AI is involved. It can be beneficial for creating ML/DL-based apps that deal with private information, like those in the healthcare, biometrics, and financial sectors. By keeping user data private, ML models can guarantee the system’s smooth operation and win over users’ confidence. As a result, AI-assisted MWI systems that respect users’ confidentiality need to be created^167^.

## Conclusion

In conclusion, the dynamic MWI and AI represent a transformative force in modern healthcare. The paper systematically navigated the landscape of this synergy, emphasizing the pivotal role played by AI algorithms in advancing the capabilities of MWI techniques. The comparative analysis of prior works showcased the multifaceted applications of AI in both MWI for healthcare and the broader context of AI assistance in MWI. This comprehensive exploration not only highlighted the current state-of-the-art technology in MWI but also illuminated its historical evolution, providing a contextual backdrop for understanding the trajectory of intelligent systems within this domain. As we delve into the extensive examination of prominent works, it becomes evident that while substantial progress has been made, challenges persist. The critical evaluation of these challenges, such as generalization to diverse conditions, underscores the need for robust solutions to ensure the reliability of AI-assisted MWI systems in varied clinical environments. Recognizing these obstacles as opportunities for improvement, the paper advocates for continued research and innovation to overcome the existing limitations and pave the way for more resilient and adaptable systems. Looking ahead, the paper not only celebrates the current advancements but also ventures into the realm of future possibilities. In essence, the intersection of MWI and AI not only signifies the present state of innovation but also holds the promise of a continually evolving and improved healthcare paradigm.
